# Comparative genome analysis identifies two large deletions in the genome of highly-passaged attenuated *Streptococcus agalactiae* strain YM001 compared to the parental pathogenic strain HN016

**DOI:** 10.1186/s12864-015-2026-y

**Published:** 2015-11-04

**Authors:** Rui Wang, Liping Li, Yan Huang, Fuguang Luo, Wanwen Liang, Xi Gan, Ting Huang, Aiying Lei, Ming Chen, Lianfu Chen

**Affiliations:** Guangxi Key Laboratory for Aquatic Genetic Breeding and Healthy Aquaculture, Guangxi Academy of Fishery Sciences, Nanning, 530021 People’s Republic of China; Guangxi Center for Disease Control and Prevention, Nanning, 530028 People’s Republic of China; Liuzhou’s Aquaculture Technology Extending Station, Liuzhou, 545006 People’s Republic of China; Institute of Applied Mycology, Huazhong Agricultural University, Wuhan, 430070 People’s Republic of China

**Keywords:** *Streptococcus agalactiae*, Genetic variation, Virulence attenuation, Sequence analysis, Virulence factors

## Abstract

**Background:**

*Streptococcus agalactiae* (*S. agalactiae*), also known as group B *Streptococcus* (GBS), is an important pathogen for neonatal pneumonia, meningitis, bovine mastitis, and fish meningoencephalitis. The global outbreaks of *Streptococcus* disease in tilapia cause huge economic losses and threaten human food hygiene safety as well. To investigate the mechanism of *S. agalactiae* pathogenesis in tilapia and develop attenuated *S. agalactiae* vaccine, this study sequenced and comparatively analyzed the whole genomes of virulent wild-type *S. agalactiae* strain HN016 and its highly-passaged attenuated strain YM001 derived from tilapia.

**Methods:**

We performed Illumina sequencing of DNA prepared from strain HN016 and YM001. Sequencedreads were assembled and nucleotide comparisons, single nucleotide polymorphism (SNP) , indels were analyzed between the draft genomes of HN016 and YM001. Clustered regularly interspaced short palindromic repeats (CRISPRs) and prophage were detected and analyzed in different *S. agalactiae* strains.

**Results:**

The genome of *S. agalactiae* YM001 was 2,047,957 bp with a GC content of 35.61 %; it contained 2044 genes and 88 RNAs. Meanwhile, the genome of *S. agalactiae* HN016 was 2,064,722 bp with a GC content of 35.66 %; it had 2063 genes and 101 RNAs. Comparative genome analysis indicated that compared with HN016, YM001 genome had two significant large deletions, at the sizes of 5832 and 11,116 bp respectively, resulting in the deletion of three rRNA and ten tRNA genes, as well as the deletion and functional damage of ten genes related to metabolism, transport, growth, anti-stress, etc. Besides these two large deletions, other ten deletions and 28 single nucleotide variations (SNVs) were also identified, mainly affecting the metabolism- and growth-related genes.

**Conclusions:**

The genome of attenuated *S. agalactiae* YM001 showed significant variations, resulting in the deletion of 10 functional genes, compared to the parental pathogenic strain HN016. The deleted and mutated functional genes all encode metabolism- and growth-related proteins, not the known virulence proteins, indicating that the metabolism- and growth-related genes are important for the pathogenesis of *S. agalactiae*.

**Electronic supplementary material:**

The online version of this article (doi:10.1186/s12864-015-2026-y) contains supplementary material, which is available to authorized users.

## Background

*S. agalactiae,* also known as GBS, is a Gram-positive bacterium that not only causes pneumonia and meningitis in neonates, but also induces bovine mastitis and infects reptiles, amphibians, and various fishes [1–3]. With the advances in sequencing technology and the reduction of cost, the genomes of *S. agalactiae* strains of different hosts and subtypes are revealed gradually. To date, 13 complete genome sequences, 19 draft genome sequences, and 282 contig sequences of *S. agalactiae* have been made publicly available. Studies showed that the genome of *S. agalactiae* can be divided into core genome, dispensable genome, and unique genome; the dispensable genome is important for the analysis of virulence differences and the development of broad-spectrum vaccines [4, 5]. Comparative genome analysis between bacterial strains that are greatly different in host specificity or virulence may help to rapidly screen for dispensable genes, gene deletions or mutations, and differentially-expressed proteins; it is also an effective way of studying the mechanisms of cross-host infection, pathogenicity, and immunogenicity of *S. agalactiae* [6–8].

Pridgeon et al. successfully generated an attenuated *S. agalactiae* strain 138spar from tilapia-derived *S. agalactiae* serotype Ib strain 138P in laboratory using a sparfloxacin resistance strategy; comparative genome analysis indicated that *S. agalactiae* 138spar had 22 deletions larger than 6 bp and 26 SNVs [7, 9]. Although *S. agalactiae* serotype Ib strain can cause infection and diseases in various fishes and amphibians, there is no report of its pathogenicity to humans, and comparative genome and phylogenetic studies indicate that *S. agalactiae* serotype Ia and Ib are distantly related [6, 10]. Currently, *S. agalactiae* Ia is the dominant strain causing infections and deaths in a large number of tilapia in Asia, which is also the important pathogen of early-onset neonatal meningitis [10, 11]. Comparative genome studies have demonstrated that tilapia- and trout-derived *S. agalactiae* type Ia strains and human-derived strains causing neonatal meningitis have a close genomic relationship [5, 6]. Our laboratory highly passaged the tilapia-derived wild-type strongly-virulent *S. agalactiae* Ia strain HN016 and obtained the attenuated strain YM001. To study the molecular mechanisms of *S. agalactiae* pathogenicity, we performed whole-genome sequencing and comparative genome analysis with HN016 and YM001 strains and found that YM001 genome had significant variations compared to HN016; in YM001 genome, there were deletions of multiple genes related to metabolism, transport, and growth. These results are of a great reference value for unraveling the pathogenesis and developing attenuated vaccine of *S. agalactiae*.

## Results and discussion

### Whole genome alignment between S. agalactiae HN016 and YM001

The assembling result indicated that the genome size of *S. agalactiae* YM001 was 2,047,957 bp, with a GC content of 35.61 % (GenBank accession number, CP011326), while the genome size of *S. agalactiae* HN016 was 2,064,722 bp, with a GC content of 35.66 % (GenBank accession number, CP011325). The similartity between both genomes was 99.69 %. Further analysis indicated that the YM001 genome contained 2044 genes and 88 RNAs, while the genome of HN016 had 2063 genes and 101 RNAs. The genome of YM001 varied significantly compared to HN016; in addition to two large deletions of 5832 and 11,116 bp respectively (see Additional file 1: Table S1; Additional file 2: Table S2), there were another ten small deletions and 28 SNVs (see Additional file 3: Table S3; Additional file 4: Table S4; Table 1).

**Table 1:** YM001-specific genetic variations compared to HN016

YM001 position	HN016 position	Gene product	Biological function	Variation	Effect on YM001 coding
Carbohydrate metabolism					
136826	142595	Fructose-bisphosphate aldolase	Fructose and mannose metabolism	SNV	K259N substitution
780441	786056	Phosphoenolpyruvate carboxylase	Microbial metabolism in diverse environments and Carbon metabolism	SNV	I181I substitution
868738	874444	UDP-N-acetylglucosamine 1-carboxyvinyltransferase	Biosynthesis of the bacterial cell wall and is critical for bacterial survival	SNV	H126R substitution
1403613	1420411	glycosyl transferase family 8	glycosyl synthesis	SNV	T383I substitution
1534895	1551880	metallophosphoesterase	hydrolysis of phosphate	SNV	Y64C substitution
Lipid metabolism					
1805677	1822388	Phosphatidate cytidylyltransferase	Glycerophospholipid metabolism	SNV	P239L substitution
Nucleotide metabolism					
83930	89762	DNA-directed RNA polymerase subunit alpha	Primary transcript RNA production and RNA chains construction	SNV	V258A substitution
192012	197717	DNA-directed RNA polymerase subunit beta	Primary transcript RNA production and RNA chains construction	SNV	P360A substitution
1515764	1532749	Thymidylate kinase	dTDP Biosynthesis	SNV	D35D substitution
Amino acid metabolism					
2033153	2049866	Arginine deiminase	Acid tolerant	SNV	V362I substitution
Environmental information processing					
1858831	1875542	Sensor histidine kinase	Peptidoglycan metabolism	SNV	R126H substitution
Translation					
393841	399503	Transcription elongation factor NusA	RNA polymerase-associated protein	SNV	E116G substitution
407665	413327	FUR family transcriptional regulator	Peroxide stress response regulator	SNV	S42I substitution
755561	761176	S1 RNA-binding protein	Post-transcriptional control of RNAs	SNV	R460S substitution
949470	955242	chloramphenicol acetyltransferase	Hexapeptide repeat-containing transferase	SNV	G43Y substitution
1264942	1270622	TetR family transcriptional regulator	Transcriptional regulator	SNV	W122R substitution
1967217	1983929	Arginine repressor ArgR	Transcriptional regulator of arginine metabolism	SNV	117-aa C-terminal deletion
DNA repair and recombination proteins					
1022277	1028049	DNA topoisomerase I	Regulation of supercoiling and maintenance of genetic stability	SNV	T644K substitution
Transport					
253283	258943	amino acid ABC transporter permease	Membrane transport	SNV	G147E substitution
297760..297761	303421	PTS system transporter subunit IIC	Starch and sucrose metabolism	Deletion	10-aa C-terminal extension,102-aa C-terminal deletion
364407	370068	MarR family transcriptional regulator	Regulate multiple antibiotic resistance and the oxidative stress response	SNV	H106N substitution
970865	976637	Sugar ABC transporter permease	simple sugar transport system permease protein	SNV	T5T substitution
1635394	1652379	Multidrug transporter	Drug efflux proteins	SNV	M126V substitution
1663151	1680136	Glycerol uptake facilitator protein	glycerol-uptake facilitator	SNV	I44I substitution
1761614	1778325	PTS system ascorbate-specific transporter subunit IIC	Microbial metabolism in diverse environments	SNV	D327J substitution
1828308	1845019	PTS system transporter subunit IIB	Galactose metabolism	SNV	M1T substitution
2020460	2037173	Cobalt transporter ATP-binding subunit	energy-coupling factor transport system ATP-binding protein	SNV	E79K substitution
Folding, sorting and degradation					
743279..743280	748941	Recombinase RecF	Manipulate the structure of genomes	Deletion	6-aa C-terminal extension,855-aa C-terminal deletion
Unknown function					
1271532..1271533	1277212	Hypothetical protein	Unknown function	Deletion	Y63N substitution, 5-aa C-terminal extension,81-aa C-terminal deletion

### Analysis of the damages and gene deletions caused by large fragment deletion

**Fig. 1:**
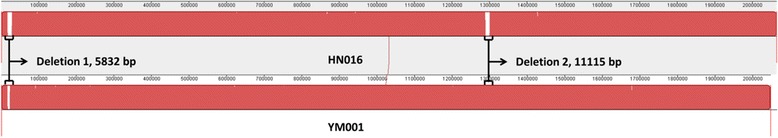
Whole genome alignment between *S. agalactiae* HN016 and YM001. The genomes of HN016 and YM001 were compared with each other using progressive MAUVE with default parameters. The colinearity of the genomes and the two deletions between HN016 and YM001 are shown

As shown in Fig. 1, YM001 genome had two large deletions compared to HN016. The deletion one was a 5832-bp sequence, which contained a repetitive sequence of 5621 bp separated by a 211-bp fragment in the genome of HN016. There were two repetitive sequences in the genome of HN016, whereas only one repetitive fragment left in the genome of YM001. This repetitive sequence contained 5S rRNA, 16S rRNA, and 23S rRNA genes, as well as other ten different tRNA genes (see Additional file 1: Table S1). The deletion two was a 11,116-bp sequence, which resulted in a truncation of two genes and deletion of 8 genes (see Additional file 2: Table S2); they were four genes of the ABC transporter family, MarR family transcriptional regulator, Ser/Thr protein phosphatase (STP), peptide deformylase, glutamate dehydrogenase, membrane protein of unknown function, and acetyltransferase.

### The deletion or damage of ABC transporter gene

ABC transporters are integral membrane proteins that conduct transmembrane transport of various solute biomolecules using the energy of ATP hydrolysis; the substances absorbed include nutrients and osmoprotectants that range from small sugars, amino acids, and small peptides to metals, anions, iron chelators (siderophores), and vitamin B12, while the exported substances are surface components of the bacterial cell (such as capsular polysaccharides, lipopolysaccharides, and techoic acid), proteins involved in bacterial pathogenesis (such as hemolysin, heme-binding protein, and alkaline protease), peptide antibiotics, heme, drugs, and siderophores [12, 13]. Among the four ABC transporter family genes deleted in the YM001 genome, three belong to the subfamily B with efflux function, two of which encode proteins that have been demonstrated relating to the efflux of various drugs, to help bacteria obtain multidrug resistance to several antibiotics and tolerance to biocides [14, 15]; the 4th one encodes a protein of subfamily F, which involves in the intercellular communication between bacteria, and the deletion of this gene may cause growth inhibition of the mutant [16]. Comparative genome analysis of *Mycoplasma hyopneumoniae* (*M. hyopneumoniae*) pathogenic 168 strain and its highly-passaged attenuated strain 168 L showed that the ABC transporter proteins might affect the growth and survival of *M. hyopneumoniae* in different hosts or host tissues [17]. The growth of YM001 in both solid and liquid cultures was significantly slower than that of HN016 [18]. Fluorescent quantitative detection of tissue bacteria after oral gavage of tilapia with the two strains respectively also indicated that both the survival time and number of HN016 *in vivo* in tilapia were significantly greater than those of YM001 [18]. A decreased growth performance of YM001 due to ABC transporter deletion might be one of the main reasons of reduced virulence.

### The deletion of MarR family transcriptional regulator

Oxidative, nitrosative, and aerobic stresses are major factors affecting the survival of pathogens in the host [19]. *S. agalactiae* is a facultative anaerobe, with a wide range of hosts, and may colonize in many tissues including the gastrointestinal and genitourinary tracts, brain, blood, liver, kidney, mammary gland, etc. [20]. The wide host range and colonization tissues of *S. agalactiae* may be associated with the ability of its regulatory systems to sense and adapt to external stimuli, such as oxidative and aerobic stress. Studies have shown that oxygen affects the infectivity and virulence of *S. agalactiae* [21]. MarR family transcriptional regulator has the function of regulating the oxidative stress response; therefore, deletion of MarR coding genes may result in an increased bacterial sensitivity to oxidative and aerobic stress, decreased capability of intracellular survival in macrophages, and reduced virulence [22]. The ability of *S. sgalactiae* to survive in macrophages is an important mechanism for its escape from the host immunity [23]. Accordingly, we speculate that the deletion of MarR family transcriptional regulator coding genes in YM001 reduced its growth adaptability and ability to survive in macrophages, decreased its ability to escape from the immune defenses of tilapia, and thereby blocked its continuous growth in and pathogenicity to tilapia.

### The deletion of STP

**Fig. 2:**
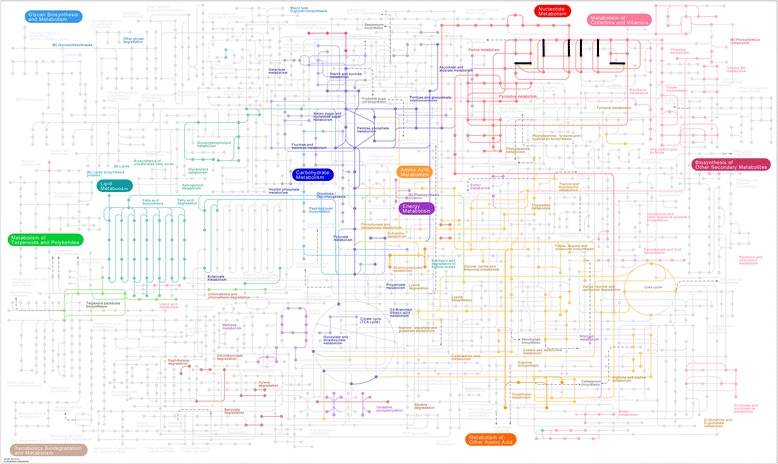
Metabolic potential. The metabolic pathways of *S. agalactiae* strains HN016 and YM001 were mapped and analyzed using KEGG Pathway Database. Those pathways, containing mutations affected metabolic-related genes, are shown in thick black line

Protein phosphorylation is essential for the regulation of cell growth, division, and differentiation in both prokaryotes and eukaryotes. Lately, bacterial homologues of eukaryotic STP have been shown to be necessary for cellular functions such as growth, differentiation, pathogenicity, and secondary metabolism. Mutations in these genes exhibited pleiotropic effects on the growth, virulence, and cell segregation of *S. sgalactiae,* suggesting that these enzymes may regulate the pyrophosphatase activity and other cellular functions in *S. sgalactiae* [24], and that these genes may have novel roles in regulating bacterial metabolic processes such as purine biosynthesis [25]. The deletion of STP in YM001 resulted in the loss of multiple purine metabolic pathways (Fig. 2). In *S. sgalactiae*, STP controls the function of Ser/Thr kinase, post-transcriptional regulation of hemolysin, autolysis, and virulence. Although STP is not essential for growth, it is critical for the pathogenicity of *S. sgalactiae* [26]. In view of its important roles in metabolism and pathogenicity of *S. agalactiae*, STP deletion may be one of the main causes of the reduced virulence of YM001.

### The deletion of the other four genes

The other four deleted genes were peptide deformylase (PDF), glutamate dehydrogenase (GDH), acetyltransferase, and a membrane protein of unknown function. PDF is a highly conserved metalloprotease for bacterial growth and participates in bacterial protein biosynthesis and maturation [27, 28]; blocking of its function significantly inhibits the growth of Gram-positive pathogens, such as *Streptococci* and *Staphylococci* [29]. GDH is an important functional molecule in the process of energy metabolism in bacteria and is related to bacterial pathogenicity; it has been identified as a virulence factor of *S. suis* serotype two [30]. PDF and GDH were deleted from the genome of YM001, which might be the cause of the slow growth and low virulence of YM001. The effect of the deletions of acetyltransferase and the membrane protein of unknown function on *S. agalactiae* remains to be further studied.

### SNV and Indels analyses between YM001 and HN016 genomes

SNV and Indels analyses between YM001 and HN016 genomes indicated that in addition to the two large deletions, there were a total of 28 SNVs (of which 1 was located in the non-coding region), and relative to HN016, YM001 had another ten deletions (including seven located in the non-coding region). Among the 27 SNVs in the coding region, 24 encoded proteins related to metabolism (ten genes), translation (six genes), and transport (eight genes) (Table 1). Two of the three deletion mutant Genes in YM001 coded for PTS system transporter subunit IIC and recombinase RecF respectively, while the third one encoded a hypothetical protein of unknown function. However, currently known *S. agalactiae* virulence factors such as adhesin, exoenzyme, immunoreactive antigen, metal transport, protease, toxin, etc., the majority of which are considered main antigens of *S. agalactiae,* did not show any variation [5]. These results indicated that genetic changes in attenuated strain generated by continuous passaging mainly affected genes related to bacterial growth and metabolism, with little effect on the virulence-related genes, which is thus conducive to the preservation of antigenicity during virulence attenuation by passaging. This may be why attenuated YM001 retained its strong immunogenicity. Current development of attenuated *S. agalactiae* vaccine mainly focused on the modification of its virulence factors; however, the results of this study opened a new avenue to the development of attenuated vaccine, i.e., to produce attenuated strain through modifying growth-related genes, under the premise of maximally preserving its immunogenicity and not affecting the virulence factors.

### Clustered regularly interspaced short palindromic repeats (CRISPRs)

**Fig. 3:**
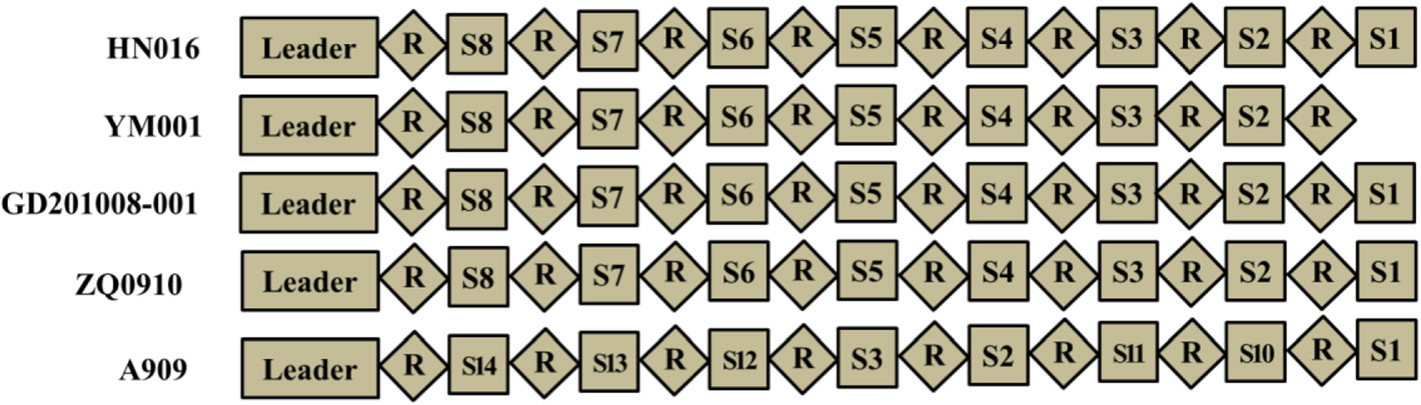
Diversity of the CRISPR1 locus in 5 *S. agalactiae* strains. Spacers were identified by the CRISPRtionary program, with numbers assigned to each spacer [51]. The names of strains are given on the left. R stands for Repeat, and S stands for Spacer

CRISPRs are a bacterial adaptive immune defense mechanism against the invasion of foreign genes. When foreign gene invades bacteria, the CRISPRs integrate and save the intruding gene fragment. Under the re-invasion of the same genes, mediated by specific RNA, CRISPRs and CRISPR-associated proteins (Cas proteins) will cut and destroy the invading foreign genes, which may include bacterial phages, plasmids, and mobile genetic elements (MGEs) [31, 32]. *S. agalactiae* has 2 CRISPR/Cas systems, type 1-C CRISPR2 and type 2-A CRISPR1; while the latter is ubiquitous, the former is only present in a few strains [33]. The CRISPR sequences were analyzed among the 8 *S. agalactiae* strains in Table 2 using the CRISPRs web server (http://crispr.u-psud.fr/Server/). The results indicated that 3 tilapia-derived *S. agalactiae* serotype Ib strains did not contain any CRISPR sequences, while 5 *S. agalactiae* serotype Ia strains all had CRISPR1 but did not contain CRISPR2. Further analysis of the CRISPR1 from the 5 *S. agalactiae* serotype Ia strains showed that the CRISPR sequence in *S. agalactiae* strain HN016 derived from tilapia in China was same as that in GD201008-001 and ZQ0910 and had eight spacers, but the attenuated strain YM001 only contained seven spacers; all other sequences were the same between both strains (Fig. 3). During the process of foreign nucleic acid invasion and bacterial evolution, to avoid overly long locus of CRISPRs, bacteria may choose to insert or remove spacer sequences between CRISPRs, and the insertion or removal of spacer is polarized, i.e., a new spacer is always inserted between the leader sequence and the following repetitive sequence, while the removed spacer is usually located at the 3’ end of CRISPRs [34]. Lopez-Sanchez et al. analyzed the CRISPRs of more than 200 wild-type *S. agalactiae* strains but did not find the 3’ terminal deletion [33]. Although Liu et al. showed that two spacer sequences were deleted at the 3’ end of CRISPRs in ZQ0910 stain [12], we found that the assembling of the published sequence of this fragment in ZQ0910 had certain mistake. After reanalysis and alignment of this sequence, we confirmed that the CRISPRs of ZQ0910 were exactly the same as those of HN016 and GD201008-001. Compared to other tilapia-derived wild-type virulent strains, the CRISPRs of YM001 had a deletion of 1 spacer at the 3’ end. Philippe et al. studied the CRISPR loci of *S. thermophilus* and found that the selective removal of spacer sequence may be caused by that these spacer sequences have little value for the survival of bacteria in the environment at the time [35]. Therefore, normal natural growth and passage are hard to cause the removal of CRISPR spacer sequence in *S. agalactiae*, whereas in the absence of the threat of foreign nucleic acid invasion, highly intensified continuous passage in laboratory may lead to the loss or removal of spacer sequence in CRISPRs.

**Table 2:** Characteristics of sequenced *S. agalactiae* strains used in this study

Strain	Serotype	MLST types	Accession	Status	Size (Mb)	Number of genes	Number of proteins	Isolatehost	Origin	Virulence description
			No.							
HN016	Ia	ST-7	CP011325	Complete	2.065	2063	1943	Tilapia	China	virulent
YM001	Ia	ST-7	CP011326	Complete	2.048	2044	1929	Tilapia	China	attenuated
A909	Ia	ST-7	NC_007432	Complete	2.128	2136	1996	Human	USA	virulent
GD201008-001	Ia	ST-7	NC_018646	Complete	2.063	2088	1964	Tilapia	China	virulent
ZQ0910	Ia	ST-7	NZ_AKAP00000000	Scaffold	2.035	2003	1970	Tilapia	China	virulent
138P	Ib	unknown	CP007482.1	Complete	1.839	1831	1593	Tilapia	USA	virulent
138spar	Ib	unknown	CP007565.1	Complete	1.838	1825	1590	Tilapia	USA	attenuated
SA20-06	Ib	ST-553	NC_019048	Complete	1.821	1872	1710	Tilapia	Brazil	virulent

### Prophages

**Fig. 4:**
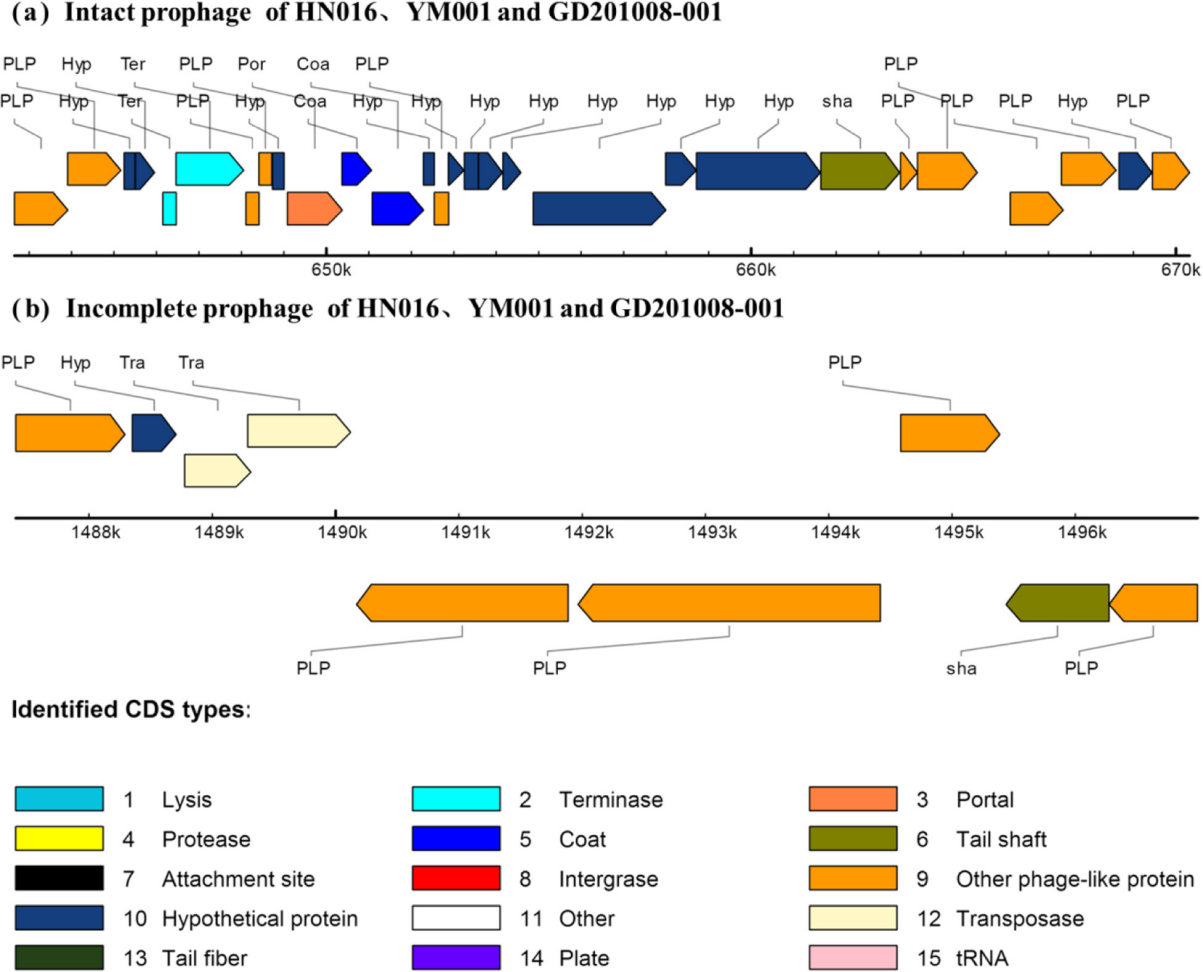
The CDS of the prophages derived from *S. agalactiae* HN016, YM001, and GD201008-001 respectively. A detailed view of the prophages from the 3 strains was produced using the online software PHAST (http://phast.wishartlab.com/index.html). Different colors represent various phage elements

Prophages are bacterial phages that integrate their genomes into the genome of host bacteria after infection. Approximately 65 % of the completely sequenced bacterial genomes carry prophage, and the content of prophage sequences in some bacteria approaches 20 % of the bacterial genomic content [36]. Studies have reported that whether a bacterial genome carries prophage often determines the difference in virulence between pathogenic and nonpathogenic strains [37, 38]. The contribution of prophage genes to the pathogenicity of *S. enterica* serovar Typhimurium has been demonstrated by animal experiments [39]. Prophage analysis of HN016, YM001, and GD201008-001 showed that an intact prophage sequence and an incomplete prophage sequence were detected in all the 3 strains. All coding DNA sequences (CDSs) and the locations of the prophages in these three strains were exactly the same (Fig. 4). Our results showed that there was no prophage mutations in the attenuated *S. agalactiae* strain YM001, which may also help to maintain the antigenic integrity of the attenuated strain.

## Conclusions

In summary, compared to the parental pathogenic strain HN016, the genome of attenuated *S. agalactiae* YM001 showed significant variations, resulting in the deletion of 10 functional genes, which may be the main reason for the loss of YM001 virulence to tilapia. The deleted and mutated functional genes all encode metabolism- and growth-related proteins, not the known virulence proteins, indicating that the metabolism- and growth-related genes are important for the pathogenesis of *S. agalactiae*. The mutations in growth- and metabolism-related genes with the preservation of virulence genes reduced the virulence while retained the full antigenecity. Our results laid a foundation for the development of attenuated *S. agalactiae* vaccine and the study on the immune mechanism. Therefore, the present study set a basis for future investigation of the pathogenesis of *S. agalactiae* and facilitated the design of attenuated vaccine.

## Methods

### Bacterial strains

The *S. agalactiae* strain HN016 originally isolated in 2010 in China, from a moribund cultured tilapia with typical clinical and pathogenic characteristics of meningoencephalitis, belonged to *S. agalactiae* serotype Ia, multilocus sequence type seven (ST7) [17]. This field strain was gradually attenuated by 840 continuous passages in TSB medium, and the 840th passage was named strain YM001 [18]. The serotype, ST type, and PFGE bands of YM001 strain were consistent with those of HN016 [18]. However, the YM001 failed to cause disease or death in tilapia at the dose of 10^9^ CFU/fish by intraperitoneal injection, while the HN016 was lethal to tilapia at the dose down to 10^3^ CFU/fish [18]. Backpassage safety assay indicated that YM001 did not cause disease or death in tilapia after 11 generations of serial passage [9, 18]. The genomes of another 6 *S. agalactiae* strains were retrieved from the GenBank (Table 2). The strains GD201008-001, ZQ0910, and A909 all belonged to serotype Ia and ST7. The GD201008-001 and ZQ0910 strains were isolated from farmed tilapia in China, and the A909 was also proven closely related to GD201008-001 [5]. The serotypes of 138P, 138spar, and SA20-06 all belonged to Ib, and the 138spar was obtained by attenuation of 138P using sparfloxacin resistance strategy.

### Genome sequencing and annotation

The draft genome sequences of *S. agalactiae* strain HN016 and YM001 were determined using Illumina Genome Analyzer II (GAII) at the Beijing Genomics Institute (BGI; Shenzhen, China). Draft assemblies were based on 454-Mb reads. All reads provided about 214-fold coverage of the genome. The GAII paired-end reads were assembled with the SOAPdenovo 2.04 program [40]. Gaps were closed by primer walking and sequencing of PCR products. Putative open reading frames (ORFs) with more than 30 amino acid residues were predicted using Glimmer 3.02 [41], while rRNAs and tRNAs were identified using RNAmmer 1.2 [42] and tRNAscan-SE 1.23 [43] respectively. The scaffolds were searched against the COG (Clusters of Orthologous Groups), GO (Gene Ontology), SwissProt, and KEGG (Kyoto Encyclopedia of Genes and Genomes) databases to annotate the gene descriptions.

### Comparative genome analysis

Nucleotide comparisons and single nucleotide polymorphism (SNP) analysis for strains HN016 and YM001 were performed using the Artemis Comparison Tool (ACT) [44] and Mauve 2.3.1 genome alignment software [45]. ORF graphical visualization and manual annotation were carried out using Artemis, release 12 [46]. Screening for unusual coding differences between the HN016 and YM001 genomes (stops and frame shifts) was conducted using FASTA program packages [47, 48] and BLAST [49, 50]. The coding differences between the HN016 and YM001 genomes were checked manually.

### Genome element prediction

CRISPRdb database, CRISPRs finder, and CRISPRcompar were used to display CRISPRs, generate dictionary of spacers and repeats, and compare CRISPRs (http://crispr.u-psud.fr/) [51–53]. PHAST (http://phast.wishartlab.com/index.html) was used to identify prophage sequences [54]. Amino acid sequences of the CDSs of 4 piscine strains and Ia strain were searched against the Virulence Factor of Pathogenic Bacteria database (VFDB, www.mgc.ac.cn/VFs/main.htm) using BLASTp [55–57]. An E value cut-off of 1e-5 was used to obtain the single best hit.

### Accession numbers

The genome sequences of *S. agalactiae* strains HN016 and YM001 were deposited into the GenBank under the accession numbers of CP011325 and CP011326 respectively.

## Additional files

Additional file 1: Table S1. The large fragment deletion 1 detected in YM001 compared to HN016. (XLS 26 kb) Additional file 2: Table S2. The large fragment deletion 2 detected in YM001 compared to HN016. (XLS 28 kb) Additional file 3: Table S3. SNVs detected in YM001 compared to HN016. (XLS 34 kb) Additional file 4: Table S4. Deletions detected in YM001 compared to HN016. (XLS 28 kb)
